# Emerging concepts on one-carbon metabolism as a unique and actionable vulnerability in cancer

**DOI:** 10.1016/j.aspetd.2026.100029

**Published:** 2026-05-29

**Authors:** Mathew Schneider, Zhanjun Hou, Larry H. Matherly

**Affiliations:** 1 Department of Oncology, Wayne State University School of Medicine, Detroit, Michigan; 2 Molecular Therapeutics Program, Barbara Ann Karmanos Cancer Institute, Detroit, Michigan; 3 Department of Pharmacology, Wayne State University School of Medicine, Detroit, Michigan

**Keywords:** (Anti)folate, 5,10-Methylenetetrahydrofolate dehydrogenase, Proton-coupled folate transporter, One-carbon metabolism, Reduced folate carrier, Serine hydroxymethyltransferase

## Abstract

Metabolic reprogramming is a defining feature of cancer, and folate-mediated one-carbon (C1) metabolism occupies a central role in supporting malignant growth, survival, and immune evasion. Although cytosolic C1 enzymes have long been exploited for the therapy of a host of cancers through the use of “classical” antifolates, growing evidence suggests that mitochondrial C1 metabolism is a critical driver of tumor progression, metastasis, and redox homeostasis. Mitochondrial serine catabolism via serine hydroxymethyltransferase 2 and 5,10-methylene tetrahydrofolate dehydrogenase 2 provides formate, glycine, and reducing equivalents to sustain nucleotide and amino acid biosynthesis, biological methylation reactions, and antioxidant defenses. Dysregulation of mitochondrial C1 metabolism promotes “formate overflow”, alters tumor–immune interactions, and supports metastatic signaling. This review synthesizes recent key advances in folate biology, and compartmentalized C1 metabolism, leading to the development of an exciting new generation of cytosolic- and mitochondrial-targeted therapies. We examine emerging links between C1 metabolism and the tumor microenvironment, including relationships between C1 metabolism and immune suppression, T cell infiltration, and macrophage polarization. Finally, we highlight critical unresolved questions and vexing future challenges in implementing the next generation of C1-targeted anticancer agents. Collectively, these insights underscore the remarkable biology of C1 metabolism, and mitochondrial C1 metabolism in paticular, and its potential as a multifaceted and therapeutically actionable vulnerability in cancer.

**Significance Statement:**

This minireview summarizes recent advances in folate biology and one-carbon (C1) metabolism. Topical areas include mechanisms of membrane transport, compartmentalization of C1 metabolism and its role in tumor biology, and the relationship between C1 biology and the tumor microenvironment. The goal of this study is to provide an evolving framework for discovery of tumor-specific vulnerabilities in C1 metabolism that can be exploited for therapy.

## 1. Introduction

Metabolic reprogramming is a hallmark of cancer^1–3^ for which one-carbon (C1) metabolism is especially notable. C1 metabolism is a paradigm of altered metabolism in cancer that offers a remarkable array of potential therapeutic targets.^4–7^ Cytosolic C1 enzymes (eg, dihydrofolate [DHF] reductase [DHFR] and thymidylate synthase [TS]) were identified decades ago as vulnerabilities leading to the discovery of an array of antifolate drugs that continue to be used today in the therapy of a growing number of cancers.^6,7^ In recent years, there has been increasing focus on the therapeutic potential of targeting C1 metabolism in mitochondria given its importance to the malignant phenotype including tumor progression and metastasis, its relationship to the tumor microenvironment (TME), and as a primary source of C1 units and glycine, as well as reducing potential.^4,5,8–12^ Growing evidence suggests that serine hydroxymethyltransferase 2 (SHMT2) and 5,10-methylene (me) tetrahydrofolate (THF) dehydrogenase 2 (MTHFD2) (respectively, the first and second enzymes in the mitochondrial C1 pathway, Fig. 1) are bona fide tumor “drivers” and promising therapeutic targets for an increasing number of cancers.^13^ Indeed, mitochondrial C1 metabolism is assuming a broader role in tumor biology, with disease progression and cancer metastasis accompanied by “formate overflow” in mitochondria as a causal factor.^8,9^ In this minireview, we discuss emerging concepts and critical unanswered questions related to the clinical impact of C1 metabolism in general, and mitochondrial C1 metabolism in particular, including their relationships to the oncogenic phenotype and the TME, and the immense therapeutic potential of targeting these pathways.

## 2. Folate biology and one-carbon metabolism in cancer

Folates are hydrophilic anions at physiologic pH that cross biological membranes poorly by diffusion. Accordingly, mammalian cells have evolved sophisticated transport systems for the uptake of folates across plasma membranes.^14–20^ Folate uptake is principally mediated by the major facilitative folate transporters SLC19A1 (reduced folate carrier [RFC])^16,17^ and SLC46A1 (proton-coupled folate transporter [PCFT]) (Fig. 1).^17,19^ The biology and structures of both RFC and PCFT, including their physiologic and disease relevance have been extensively reviewed.^6,14–19^

Folate uptake can also be mediated by folate receptor (FR) proteins that are expressed as different isoforms with distinct tissue and cell patterns of expression.^20–22^ FR*α* is expressed on the surface of a number of tumors (eg, non-small cell lung cancer, triple-negative breast cancer, and endometrial cancer) and a number of tissues (kidney, lung, choroid plexus, and placenta),^20–22^ whereas FR*β* is expressed in tumor-associated macrophages (TAMs) and myeloid-derived suppressor cells (MDSCs).^23,24^ However, FRs are not a major mechanism of folate uptake. Folate efflux from mammalian cells primarily involves multidrug resistance-associated proteins (eg, ABCC1–5) and ABCG2.^25,26^

Folates are transported from the cytosol into mitochondria, reportedly by SLC25A32 localized to the inner mitochondrial membrane,^27^ although a role of SLC25A32 in FAD transport has also been suggested.^28,29^ *SLC25A32* gene amplification accompanies poor prognoses in ovarian and breast cancers.^29^ In glioblastoma cells, SLC25A32 promotes tumor cell growth and invasion, as well as the phosphatidylinositol 3-kinase/protein kinase B signaling,^30^ suggesting a more complex role in malignancy.

Both cytosolic and mitochondrial folates are metabolized to other folate forms differing in their redox character (DHF and THF] and their association with C1 substituents (eg, methyl, methylene, and formyl). Further, folates and related molecules are metabolized by folylpolyglutamate synthetase (FPGS) in both the cytosol and mitochondria, resulting in conjugation of up to 7 additional L-glutamic acid residues at the γ carboxyl of the terminal glutamate of the parent folate.^31,32^ Polyglutamate folate forms are generally preferred substrates for C1 enzymes over nonpolyglutamyl folates.^31,32^

Whereas parent “monoglutamate” folates cross membranes via bidirectional transport processes involving facilitative transporters^17^ and are substrates for anionic efflux pumps,^25,26^ folate polyglutamates are poorly transported and are selectively retained in cells compared with parent (unconjugated) folates independent of extracellular folate levels.^31,32^ Further, cytosolic folate polyglutamates do not exchange with mitochondrial folate polyglutamates.^33^ Selective deletion of mitochondrial FPGS activity impairs cellular accumulation of both cytosolic and mitochondrial folates and results in a profound loss of C1 metabolic flux in both compartments and decreased C1-dependent biosynthesis.^34^

Tumor cells are highly glycolytic and have elevated expression of glucose transporters to support their glycolytic flux.^35^ Serine is synthesized from glucose in the cytosol from 3-phosphoglycerate (a glycolytic intermediate) catalyzed by phosphoglycerate dehydrogenase (PHGDH) (Fig. 1).^5,10,11^ The PHGDH product, 3-phosphohydroxypyruvate, is transaminated by phosphoserine aminotransferase to 3-phosphoserine.^36^ 3-PS is converted to serine, catalyzed by phosphoserine phosphatase.^36^ Under serine-starved conditions, pyruvate kinase 2 (the last enzyme in glycolysis) activity is suppressed, promoting the shuttling of 3-phosphoglyerate toward serine biosynthesis.^12^ Further, excess serine regulates its own synthesis (at PHGDH).^37^ Alternatively, serine can be obtained from dietary sources or protein degradation; extracellular serine is transported into cells in a sodium-dependent manner by SLC1A4, SLC1A5, SLC38A1, or SLC38A2.^38^ Reliance on serine biosynthesis from glucose vis á vis extracellular sources varies depending on expression of PHGDH.^39^

Serine is the primary donor of C1 units for cellular biosynthesis and other C1 processes.^5^ Cytosolic serine is actively transported into the mitochondria by sideroflexins 1 (SFXN1, *SLC56A1*) and −3 (SFXN3, *SLC56A3)* (Fig. 1).^40^ SFXN1 is regulated by *Myc* and is highly expressed in primary tumors and tumor cell lines.^40,41^ SFXN3 is also expressed in tumors, whereas SFXN2 levels are typically very low.^40^ Thus, SFXN1 and SFXN3 are potential determinants of mitochondrial serine levels and mitochondrial C1 flux in tumors. After its transport into the mitochondria, serine is converted to glycine by SHMT2, accompanying conversion of THF to 5,10-me-THF. This is followed by the metabolism of 5,10-meTHF to 10-formyl THF and NADH by MTHFD2 (or NADPH by MTHFD2-like (MTHFD2L) in normal tissues].^13,42,43^ Further, 10-formyl THF is metabolized to formate and THF by MTHFD1L (5,10-me-THF dehydrogenase 1-like) with the generation of ATP. Thus, the C3 of serine is ultimately metabolized to formate, which is transported into the cytosol.^10–12,44–46^

Mitochondrial 10-formyl THF is also a substrate for aldehyde dehydrogenase 1 family member L2 (ALDH1L2), generating NADPH, CO_2_ and THF (Fig. 1).^47,48^ The metabolic role of ALDH1L2 is less characterized than its cytosolic homolog aldehyde dehydrogenase 1 family member L1 (ALDH1L1),^48^ although ALDH1L2 in mitochondria is overexpressed in several tumors^12,13^ and has been associated with a poor prognosis for a number of cancers, including colorectal cancer and pancreatic ductal adenocarcinoma.^48–50^ ALDH1L2 is also an important source of mitochondrial NADPH that serves an essential antioxidant role to limit the accumulation of reactive oxygen species (ROS) in hypoxic tumors.^5,51^ Importantly, ALDH1L2 channels C1 units from 10-formyl THF to CO_2_ at the expense of mitochondrial formate,^47^ potentially limiting cytosolic C1 units for cellular biosynthesis of nucleotides and synthesis of mitochondrial formyl methionine required for translation of mitochondrial proteins.^47^ Loss of ALDH1L2 also results in increased generation of mitochondrial formate, stimulating tumor metastasis (via formyl-peptide receptor-dependent signaling)^47,52^ and immune cell activation.^47^

In the cytosol, 10-formyl THF is resynthesized from THF and formate from mitochondria by the 10-formyl THF synthetase activity of MTHFD1 (Fig. 1).^4,5^ The mitochondrial derived formate stabilizes cytosolic THF protecting it from oxidative damage compared with unsubstituted THF forms.^53^ MTHFD1 cyclohydrolase and dehydrogenase activities further convert 10-formyl THF to 5,10-me-THF with the oxidation of NADPH to NADP^+^. SHMT1 is a cytosolic homolog of SHMT2 that catalyzes the reverse reaction whereby glycine is converted to serine with a C1 unit derived from 5,10-me-THF.^4,5^ TS catalyzes the conversion of deoxyuridine monophosphate to dTMP with 5,10-me-THF providing both a C1 unit and a reducing equivalent, resulting in the generation of DHF. DHF is reduced back to THF by DHFR using a reducing equivalent from NADPH such that loss of DHFR activity (ie, treatment of methotrexate) results in a depletion of THF pools and suppression of THF-dependent metabolism.^7^ However, even when DHFR activity is abolished, THF pools are incompletely converted to DHF, likely reflecting folate compartmentation to mitochondria and other organelles^4,5,44^ as well as THF cofactors bound to cellular proteins.^54^

5,10-me-THF can be reduced to 5-methyl THF by 5,10-me-THF reductase.^4,5^ 5-methyl THF provides C1 units for the B12-dependent conversion of homocysteine to methionine catalyzed by methionine synthase (Fig. 1).^5,44^ Methionine is metabolized by S-adenosylmethionine synthetase to S-adenosyl methionine required for methylation of DNA, RNA, phospholipids and proteins.^5,44^

De novo purine biosynthesis involves 10 reactions from phosphoribosyl pyrophosphate to IMP (Fig. 2).^55^ Glycine is incorporated into purines in a reaction catalyzed by the trifunctional glycinamide ribonucleotide formyltransferase (GARFTase). C1 units are incorporated at the third and ninth steps by GARFTase and 5-aminoimidazole-4-carboxamide ribonucleotide (AICAR) formyltransferase (AICARFTase). To facilitate the efficient flux of pathway intermediates, de novo purine biosynthesis enzymes are assembled into a “purinosome” complex which mediates the passage of key intermediates including THF cofactors between sequential steps from phosphoribosyl pyrophosphate to AMP and GMP.^55^ Purinosomes are reported to colocalize with mitochondria to maximize the metabolic efficiency of C1-dependent purine biosynthesis.^55^

## 3. Therapeutic targeting cytosolic C1 metabolism

Reflecting the importance of C1 metabolism to cellular homeostasis and disease, “antifolate” drugs were developed to target these essential metabolic pathways and continue to be important clinically for treating a wide range of cancers (eg, leukemias, lymphomas, non-small cell lung cancer, and malignant pleural mesothelioma), as well as other conditions (eg, rheumatoid arthritis and psoriasis).^7,25^ Methotrexate and pralatrexate are both DHFR inhibitors.^7^ Pemetrexed is a TS inhibitor that also targets de novo purine nucleotide biosynthesis (ie, GARFTase and AICARFTase) as well as DHFR.^7,25^ These “classical” antifolates are all substrates for both RFC and PCFT^7,16^ and drug transport is an important determinant of their antitumor efficacies and toxicities.^16,56^ Further, these antifolates are all metabolized to polyglutamate conjugates by FPGS analogous to folates.^31^ For classical antifolates, metabolism to polyglutamate forms results in increased drug retention and enhanced inhibitory potencies toward C1 enzyme targets.^31^ Thus, tumors that synthesize higher levels of antifolate polyglutamates should be more susceptible to antifolate therapies.^31^

Cancer cells frequently have greater metabolic needs than normal differentiated cells. These increased biosynthetic demands can at least partly be met by “aerobic glycolysis” (“Warburg effect”) through which tumor cells are increasingly glycolytic even in the presence of normal oxygen tension.^57,58^ Under these conditions, glycolytically generated lactic acid is extruded by plasma membrane ion pumps (ie, monocarboxylate transporters), resulting in acidification of the extracellular environment.^57,58^

In 2011, we reported that PCFT (SLC46A1) was highly expressed together with RFC (SLC19A1) in a substantial number of cell lines derived from human solid tumors (eg, breast, liver, lung, malignant pleural mesothelioma, pancreas, prostate, and ovarian).^59^ In contrast, PCFT was undetectable in hematologic cancers (eg, leukemia)^59^ and was expressed at very low levels in most normal tissues.^14^ PCFT expression was also detected in a number of primary human tumors including non-small cell lung cancer,^60^ epithelial ovarian cancer,^61,62^ malignant pleural mesothelioma,^63^ and pancreatic cancer (*manuscript in preparation*). Notably, pemetrexed is an excellent substrate for transport by PCFT.^7,14,16,18^ For mesothelioma patients treated with pemetrexed, PCFT levels correlate with progression-free survival and overall survival.^63^

Given its potential for targeted drug delivery at acidic pH, Matherly and Gangjee explored the possibility of developing novel antifolates as antitumor agents and as *selective substrates* for PCFT membrane transport.^6,14,59–61,64,65^ This was based on the reasoning that if cytotoxic compounds could be designed that target tumors directly via PCFT with modest transport activity by RFC, these would show far greater tumor selectivity than drugs such as pemetrexed or standard-of-care anticancer agents. Further, since PCFT expression is often limited in normal tissues and most normal tissues do not experience the acidic microenvironment that is most conducive to high levels of PCFT transport,^14,19^ cytotoxic agents that are PCFT transport substrates should be uniquely selective toward tumors.

In 2010, the first PCFT-targeted therapeutics, 6-substituted pyrrolo[2,3-*d*]pyrimidine benzoyl antifolates (AGF17 and AGF23; Table 1^45,46,64–75^), were reported.^64^ These novel compounds are nominally transported by RFC and thus should be selective for tumors over most normal tissues.^64^ PCFT transport efficiency was further enhanced by replacing the phenyl side-chain in AGF17 with a 2′,5′-thiophene (AGF94,^65^ AGF150,^67^ AGF154^67^), or a 2′ fluorinated thiophene side-chain (AGF278^68^) (Table 1). Based on these structure-activity studies, a distinct pharmacophore for selective PCFT drug targeting soon emerged,^6,14,15,76^ which was validated by cryo-electron microscopy, including structures of chicken PCFT in its apo form and in complex with pemetrexed.^77^ These results provided definitive proof-of-concept validation of therapeutic targeting tumors via PCFT.

The 6-substituted pyrrolo[2,3-*d*]pyrimidine inhibitors all targeted de novo purine biosynthesis at the first folate-dependent step catalyzed by GARFTase^65,67,68,78^ and are nanomolar inhibitors of human epithelial ovarian cancer^61^ and non-small cell lung cancer^60^ cells. In vitro studies with AGF94 and AGF278 were extended in vivo with immunodeficient mice bearing human tumor cell line xenografts (HepG2, HeLa, and H460).^60,64,67^ In vivo studies with AGF94 were later extended to a novel syngeneic model of ovarian cancer (BR-Luc) and showed substantial antitumor efficacy,^79^ and to a cisplatin-resistant patient-derived xenograft of pleural malignant mesothelioma (MPM9) (*manuscript in preparation*), providing compelling “proof-of-concept” for tumor-selective PCFT targeting.

The realization that C1 metabolism is compartmentalized between the cytosol and mitochondria and that SHMT2 and MTHFD2 in mitochondrial C1 metabolism were important “drivers” of the malignant phenotype raised new challenges and opportunities for tumor therapy. Although multiple targets afforded opportunities for combination chemotherapy, a particularly exciting new development involved the discovery of tumor-selective, multitargeted antifolates capable of targeting both mitochondrial and cytosolic C1 pathways.^45,69,70^ This is described in the following section.

## 4. Targeting mitochondrial C1 metabolism in cancer

The mitochondrial C1 enzymes SHMT2 and MTHFD2 are emerging therapeutic targets for cancer,^4,5,45,46,70–72,74,75,80^ reflecting their association with disease aggressiveness and induction under hypoxia in *Myc*-transformed cells.^12,81^ SHMT2 and MTHFD2 are among the top 5 upregulated genes (of 1454 genes) from 1981 tumors (19 tumor types) compared with matched normal tissues,^13^ further suggesting their importance to the malignant phenotype.

MTHFD2 has 40% sequence identity to MTHFD1, the cytosolic MTHFD isoform.^82^ Although crystallization of MTHFD1 complexed with inhibitors^83^ identified key catalytic residues, the development of MTHFD2 inhibitors has been challenging. Studies with LY345899 (Lilly) yielded the first crystal structure of an inhibitor complexed with MTHFD2.^80^ Although LY345899 inhibited purified human MTHFD2,^80^ LY345899 was a more potent inhibitor of MTHFD1. An MTHFD2 inhibitor (DS44960156) with a tricyclic coumarin scaffold was discovered by high-throughput screening, then modified by structure-based drug design.^74^ DS44960156 was >18-fold more inhibitory toward human MTHFD2 than MTHFD1. Scaffold optimization yielded DS18561882 (Table 1), a potent MTHFD2 inhibitor (IC_50_ = 6.3 nM; ~90-fold greater than MTHFD1).^74^

More recently, monocyclic inhibitors of MTHFD2 were described (ie, TH9619, TH9028, and TH7299^72,73^; Table 1). However, these agents do not penetrate mitochondria but rather derive their antitumor effects by targeting cytosolic MTHFD1.^73^ Interestingly, inhibition of MTHFD1 resulted in “trapping” 10-formyl THF via tight binding to the enzyme, resulting in depletion of 5,10-me-THF and thymidylate pools such that antitumor activity was exacerbated by added purines (reflects feedback inhibition of de novo purine biosynthesis).^73^ An additional MTHFD2 inhibitor was discovered using computer-aided drug design based on a chalcone scaffold (compound 41; Table 1) and exhibited selectivity for MTHFD2 over MTHFD1 resulting in elevated ROS, apoptosis and antitumor activity.^75^ However, the chalcone-based inhibitor exhibited poor pharmacokinetics in vivo.^75^

The first bona fide inhibitors of mammalian SHMT2 were pyrazolopyran compounds (SHIN1 and SHIN2)^46,71^ (Table 1). These “nonclassical” C1 inhibitors are structurally distinct from folates and antifolate compounds such as pemetrexed. Although SHIN1 and SHIN2 targeted SHMT2 in mitochondria and SHMT1 in the cytosol and resulted in in vitro antitumor activity,^46,71^ SHIN1 was inactive in vivo.^46^ SHIN2 showed in vivo efficacy toward T cell leukemia at high doses.^71^ As SHIN1 and SHIN2 are not metabolized to polyglutamates, target engagement is adversely impacted by competing folates, resulting in a reversal of drug effects accompanying elevated RFC activity.^84^

Dekhne et al^45^ described a new generation of C1 inhibitors that combined structural features of 5-substituted pyrrolo[2,3-*d*]pyrimidine compounds (AGF127 and AGF136)^85^ (de novo purine biosynthesis inhibitors) with those of 5-formyl THF and 5,10-me-THF. These 5-substituted pyrrolo[3,2-*d*]pyrimidine analogs (eg, AGF291, AGF320, and AGF347; Table 1) inhibited SHMT2 in mitochondria with secondary inhibition of cytosolic targets including SHMT1, and GARFTase and AICARFTase.^45,69,70^ The lead compound AGF347 was internalized by both PCFT and RFC (PCFT > RFC) and accumulated in both the cytosol and mitochondria.^34,62,84,86^ AGF347 was extensively metabolized to AGF347 polyglutamates by FPGS isozymes in both the cytosol and mitochondria^34,83,85^ thus circumventing adverse effects of elevated cellular folates.^84^ The pentaglutamyl form of AGF347 (AGF347-Glu_5_) showed 19-fold increased potency (decreased K_i_) for SHMT2 over the parent drug.^69^ Another member of this series, AGF359 (Table 1) also inhibited TS.^62^ Downstream effects of AGF347 included suppression of mTOR (reflecting accumulation of AICAR), depletion of glutathione, and accumulation of ROS.^62,86^ Importantly, these multitargeted C1 inhibitors showed in vitro antitumor efficacies toward colorectal cancer,^45^ non-small cell lung cancer,^45^ pancreatic ductal adenocarcinoma (both wild-type and mutant KRAS),^45,73,86^ and epithelial ovarian cancer, including high-grade serous ovarian cancer.^62^ Toward cisplatin-resistant ovarian cancer cells, AGF347 was synergistic with cisplatin.^62^ AGF347 showed promising in vivo antitumor efficacy toward SKOV3 epithelial ovarian cancer xenografts^62^ and early- and late-stage MIA PaCa-2 pancreatic ductal adenocarcinoma xenografts with curative potential.^45^

## 5. Targeting C1 metabolism and the tumor microenvironment

The TME is a complex ecosystem comprised of immune cells, fibroblasts, and signaling molecules embedded in an extracellular matrix.^87^ The TME plays an important role in the pathogenesis of cancer by supporting tumor growth and enabling metastasis, while fostering the immunosuppressive conditions that contribute to therapy resistance.^87^ The TME is important for tumor progression yet is increasingly an important target for therapy.

The pyrrolo[2,3-d]pyrimidine antifolate AGF94 (Table 1) is a substrate for cellular uptake by FRs in addition to PCFT.^65^ FR*β* is expressed in TAMs and MDSCs^23,24,79^ (Fig. 1). In epithelial ovarian cancer, TAMs and MDSCs contribute to an immune-suppressive environment, which enables disease progression while evading immune detection.^88^ TAMs contribute to metastasis and angiogenesis by releasing proangiogenic factors.^89^ Thus, FR *β*-positive TAMs and MDSCs are potential targets for FR-targeted agents.^24,79^ PCFT is also expressed in M2-like TAMs.^90^ AGF94 treatment in the BR-Luc syngeneic epithelial ovarian cancer model had modest impact on M1-like (FR*β*+, CD80+) TAMs, however, there were significant decreases in M2-like (FR*β*+, Arg1+) TAMs.^79^ When the spatial landscape of the immune infiltrate was assessed by immunofluorescence staining with FR *β* and pan-macrophage F4/80+ antibodies on tumor sections, FR *ß*-positive cells were detected in untreated mice, with significantly reduced FR *ß*-positive cells with AGF94-treatment.^79^ Thus, antitumor effects of AGF94 are accompanied by significantly decreased M2-like TAMs.^79^

Cancer cells can exploit folate metabolism to limit T cell infiltration and facilitate immune escape.^91^ To evade immune surveillance, tumor cells often “reprogram” intracellular metabolic pathways and increase expression of inhibitory immune checkpoint ligands such as programmed death-ligand 1 (PD-L1).^92^ Dysregulation of folate metabolism has been shown to contribute to mechanisms of immune evasion within the TME.^91^ For example, elevated MTHFD2 expression in pancreatic cancer cells has been associated with increased PD-L1 expression, thereby enhancing resistance to immune-mediated tumor clearance.^93^ Studies of primary diffuse large B cell lymphoma specimens revealed an inverse relationship between TS expression and the levels of HLA-A and HLA-B, implicating thymidylate metabolism in impaired antigen presentation and immune escape.^94^ Notably, treatment with pemetrexed in combination with dual immune checkpoint blockade targeting PD-L1 and HLA-G, significantly enhanced T cell–dependent cytotoxicity in non-small cell lung cancer cells.^95^ This was associated with increased protein expression and glycosylation of PD-L1 and HLA-G.^95,96^ Moreover, alterations in folate-mediated C1 metabolism may also regulate T cell infiltration into tumors. Multiple studies have reported correlations between the expression of MTHFD2, SHMT2, and ALDH1L1 in cancer cells and the presence of diverse T cell subsets, including regulatory T cells, Th17, Th1, Th2, and CD8^+^ T cells.^92,97^ Collectively, these observations demonstrate that cancer cells can remodel an immunosuppressive TME through metabolic reprogramming of C1 metabolism, although the underlying regulatory mechanisms will require further investigation.

Cellular stress from exposure to cytotoxic agents results in the release of ADP/ATP from tumors, which upon hydrolysis (via CD39 and CD73 on hematopoietic and nonhematopoietic cells [eg, lymphocytes and tumors]) generates extracellular adenosine that is immunosuppressive.^98,99^ Adenosine suppresses cytotoxic effector functions of natural killer and CD8^+^ T cells and results in increased programmed cell death protein 1 expression on CD8^+^ T cells (Fig. 1). Further, adenosine inhibits Th1 CD4^+^ T cell response and polarizes myeloid cells toward M2-like macrophages and tolerogenic dendritic cells.^98,99^ Finally, adenosine enhances the proliferation of granulocytic MDSCs and regulatory T cells, affecting T cell proliferation and function. Thus, excess extracellular adenosine suppresses immune surveillance and promotes tumor survival^98,99^ such that therapies that decrease adenosine in the TME (eg, antipurine drugs) should enhance antitumor immunity.^98,99^ Interestingly, treatment of a syngeneic model of epithelial ovarian cancer (BR-Luc) with the 6-substituted pyrrolo[2,3-*d*]pyrimidine antifolate AGF94 (Table 1) increased (~50%–100%) CD3^+^ T cells with an equal impact on CD4^+^ and CD8^+^ T cells.^79^ AGF94 decreased adenosine ~59% in BR-Luc tumors, accompanied by 3.1-fold increased interferon gamma transcripts (*manuscript in preparation*), suggesting an enhanced adaptive immune response via activation of CD8^+^ T cells.

## 6. Future directions

In this review, we summarize emerging concepts in oncology relevant to optimizing the clinical impact and therapeutic potentials of C1 metabolism in general, and mitochondrial C1 metabolism in particular. Advances over the past decade in relation to these essential metabolic pathways have been immense, in part spearheaded by the advent of widely accessible high-resolution liquid chromatography–tandem mass spectrometry methods for metabolomic profiling of C1 anabolites, combined with the discovery of a new generation of novel C1 inhibitors as tool drugs and as potential clinical candidates. Below we summarize critical unanswered questions and untested concepts that remain important areas for future study in this continually evolving area.

### 6.1. What is the impact of targeting upstream pathways for serine biosynthesis and mitochondrial serine transport in combination with SHMT2 and MTHFD2 inhibitors?

Serine is the primary donor of C1 units^4,5,10,11,44^ such that processes determining cytosolic serine accumulation in tumor cells are of particular interest. Tumor cells are glycolytic and frequently have elevated glucose transport to support the glycolytic flux.^35^ PHGDH catalyzes the rate-limiting step in serine biosynthesis from glucose; its expression is elevated in tumors compared with normal tissues for a number of cancer types including breast,^39^ pancreatic,^100^ and lung^101^ cancers and its elevated expression is often associated with poorer outcomes. Loss of PHGDH activity is associated with decreased proliferation and metastasis,^39,102^ suggesting its importance as a therapeutic target for cancer. Although inhibitors of PHGDH have been developed (including competitive^103^ and allosteric inhibitors,^104^ or inhibitors that disrupt tetramer formation^105^), none of these have advanced to the clinic for cancer. After synthesis in the cytosol, serine is transported into mitochondria by SFXN1/3 transporters.^40^ Although cells lacking SFXN1/3 experience decreased mitochondrial C1 flux similar to that resulting from loss of SHMT2 or MTHFD2,^40^ there are no clinically relevant inhibitors of SFXNn proteins. Clearly, therapies targeting PHGDH and SFXNn proteins warrant further development, especially in combination with promising mitochondrial C1-targeted therapies (eg, AGF347 and SHIN2).

### 6.2. What is the impact of ALDH1L2 on C1 homeostasis in mitochondria and cytosol, and on the antitumor efficacy of mitochondrial and cytosolic C1 inhibitors?

The broader metabolic role of ALDH1L2 in C1 homeostasis and targeted therapies remains unclear. ALDH1L2 is expressed in a wide range of tumors^48^ and with minor exceptions^47,48,50^ ALDH1L2 has been understudied. Mitochondrial ALDH1L2 catalyzes the synthesis of CO_2_, NADPH and THF from 10-formyl THF^48^ generated from MTHFD2 catalysis (Fig. 1), thus “depleting” mitochondrial formate (as CO_2_) with potentially profound impacts on de novo nucleotide biosynthesis and biological methylation (eg, 5-methyl THF) reactions in the cytosol. As mitochondrial derived formate is reported to “stabilize” labile cytosolic THF forms (eg, 10-formyl THF),^53^ the metabolic impact of ALDH1L2 on THF pools could be particularly profound. ALDH1L2 is also an important source of mitochondrial NADPH that serves an essential antioxidant role and limits ROS production.^5,51^ Further, loss of ALDH1L2 increases mitochondrial 10-formyl THF pools for synthesis of formyl-methionyl transfer RNA and translation of mitochondrial-encoded proteins, while also decreasing tumor cell migration and metastatic progression (reflecting decreased formyl-peptide receptor).^47^ Certainly, the myriad relationships among mitochondrial 10-formyl-THF consumption, formate accumulation and NADPH production impacted by ALDH1L2 are important topics for future study.

### 6.3. How are folates and mitochondrial-targeted antifolates transported into mitochondria?

The mechanism by which folates and mitochondrial-targeted antifolates are transported into mitochondria remains a conundrum. As folate polyglutamates in the cytosol do not exchange with mitochondrial forms,^33^ transport of monoglutamyl folates from the cytosol into the mitochondria is essential. Although early studies reported that folates are transported from the cytosol into mitochondria by SLC25A32,^27^ more recent studies suggested that SLC25A32 is primarily an FAD transporter^28,29^ and implied that other mechanisms of folate transport may exist. Further, whereas uptake of nonclassical SHMT2-targeted antifolates (SHIN1^46^ and SHIN2^71^) involves passive diffusion,^84^ mitochondrial uptake of emerging SHMT2-targeted antifolates such as AGF347 is uncertain.^62,86^ Finally, SLC25A32 may impact tumor physiology *independent of its role in folate transport* via effects on cell signaling.^30^ Clearly, further characterization of detailed mechanisms of (anti)folate transport into mitochondria and the broader biological role of SLC25A32 will be essential to future progress in this evolving area.

### 6.4. How is formate transported from the mitochondria into the cytosol?

Formate is generated by MTHFD1L in the mitochondrial matrix then exported into the cytosol for C1 biosynthesis including biosynthesis of nucleotides and amino acids such as serine and methionine^4,5,9,11,44^ (Fig. 1). Although formate may in part exit mitochondria via passive diffusion, an inner mitochondrial membrane transporter must exist to facilitate efficient export of formate into the cytosol although this has not yet been identified. In terms of cancer, identification of this transporter takes on particular significance, as formate export from mitochondria contributes to “formate overflow,” which promotes migration and metastasis^8,9^ and may represent a potential therapeutic target.

### 6.5. Can a specific inhibitor for MTHFD2 be developed?

Both SHMT2 and MTHFD2 were identified as emerging therapeutic targets for cancer,^4,93^ reflecting their association with disease aggressiveness and induction under hypoxia in *Myc*-transformed cells.^12,81^ While promising new agents have been developed for inhibiting SHMT2 (SHIN2^71^ and AGF347^45,70^) with in vivo efficacy toward human tumor xenografts,^45,62,71^ this has not yet been achieved for MTHFD2. Important considerations include MTHFD2 homologies with MTHFD2L, which is primarily expressed in the mitochondria of normal tissues^13,42,106^ and MTHFD1 expressed in the cytosol of both tumors and normal tissues.^81,106^ Of course, an additional consideration involves the extent to which potential MTHFD2 inhibitors can penetrate mitochondria as previous iterations of potent MTHFD2 inhibitors were mitochondrial impenetrable.^73,75^ Whereas development of an MTHFD2-specific inhibitor with tumor selectivity is appealing, an additional challenge involves the compensatory cytosolic C1 flux (via SHMT1)^5^ necessitating multitargeting (including SHMT1) to prevent resistance.

### 6.6. Role of hypoxia in C1-targeted therapies

Ischemia and acidosis promote tumor progression, decrease antitumor drug activity through ion-trapping, and result in evasion of apoptosis and cell cycle arrest.^3,58,107^ Mitochondrial C1 genes (eg, *SFXN1*, *SLC25A32*, *SHMT2*, and *MTHFD2*) are regulated by *Myc* and induced by hypoxic stress.^108^ Mitochondrial C1 flux is also essential for survival of tumor cells under low oxygen levels (0.5%–4.2% O_2_)^109^ as a primary source of cellular glycine (via SHMT2)^5,44^ and NAD(P)H (via MTHFD2 and MTHFD2L) required for synthesis of glutathione, an important scavenger of ROS resulting from the production of superoxide during electron transport under low oxygen concentrations.^110^ Paradoxically, inhibition of SHMT2 by SHIN1 under hypoxia potentiated rather than inhibited tumor proliferation. This was accompanied by decreased synthesis of NADH from MTHFD2^111^ (Fig. 1) and led to accumulation of the oxidized trichloroacetic acid cycle intermediate oxaloacetate and aspartate. Elevated aspartate in turn enhanced de novo purine biosynthesis via donation of its amine group to IMP (catalyzed by adenylosuccinate synthetase) leading to the generation of AMP (Fig. 1), thus circumventing downstream effects of targeting SHMT2.^111^ Clearly additional studies of the broader impact of hypoxia on C1 metabolism and targeted C1 therapies, including multitargeted C1 inhibitors, are warranted.

### 6.7. Alternative C1 pathways and noncanonical roles of C1 enzymes

The potential impact of alternative sources of C1 units from serine including glycine, choline, sarcosine, histidine, and tryptophan in cancer cells cannot be overlooked. Although evidence suggests that the net contribution of these pathways is modest,^5,9^ alternative C1 sources may potentially contribute to resistance to C1 inhibitors. Further, the noncanonical roles of SHMT proteins should not be ignored. For instance, SHMT1 can regulate SHMT2 expression by binding to the 5′ untranslated region of SHMT2 transcripts, suggesting a novel mechanism of SHMT regulation and a potential crosstalk between cytosolic and mitochondrial serine metabolism.^112^

Clearly, C1 metabolism remains a vibrant and continually evolving area with substantial potential for future investigation with particular relevance to cancer biology and therapy.

## Figures and Tables

**Fig. 1.**
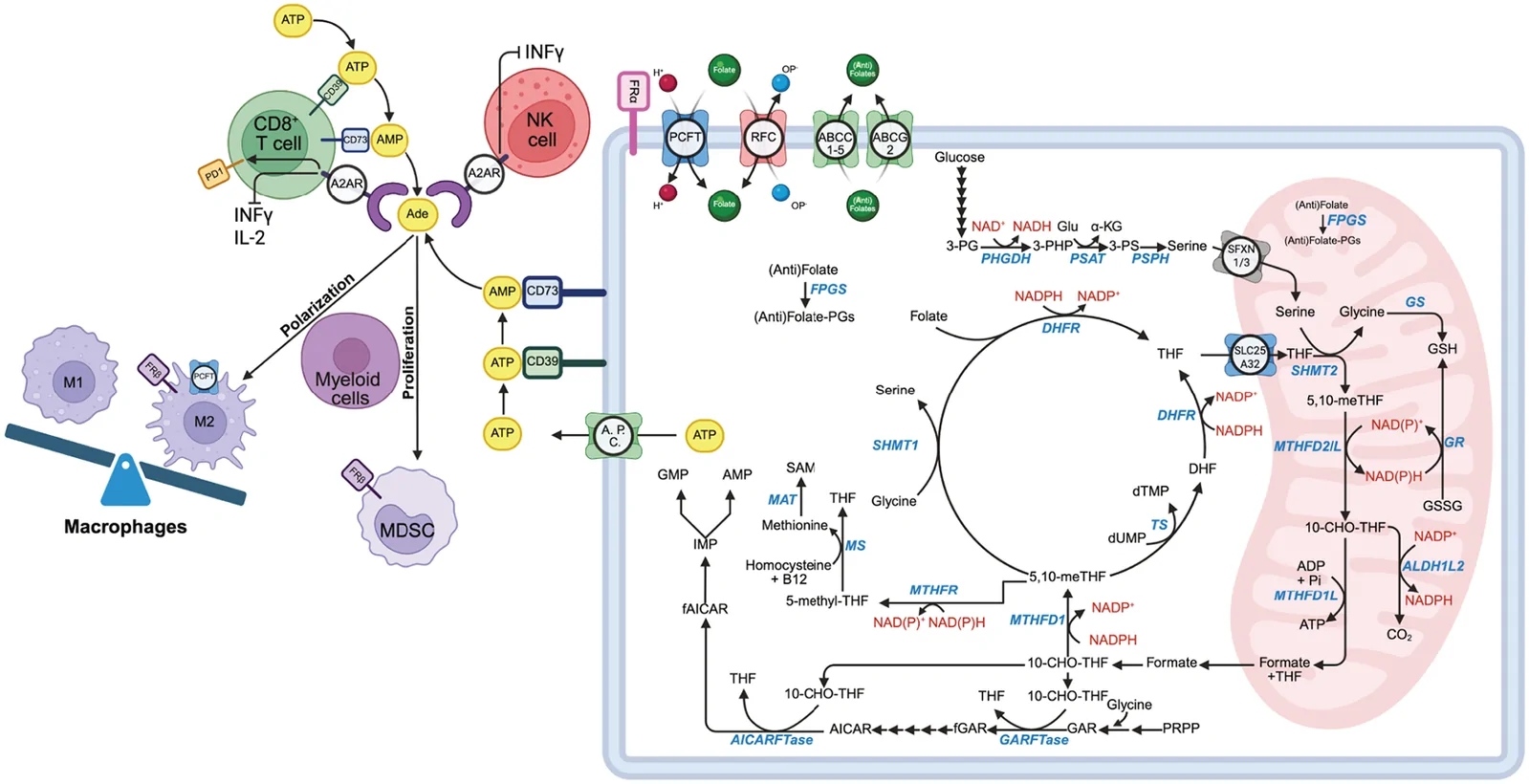
One-carbon (C1) metabolism and the TME. Folates are transported into cells by the RFC (SLC19A1) and the PCFT (SLC46A1), and by FRs and serve as cofactors for C1 metabolism. Folates are metabolized to polyglutamates by FPGS. DHF is reduced to THF by DHFR. Serine is synthesized from glucose in the cytosol from 3-phosphoglycerate (3-PG) catalyzed by phosphoglycerate dehydrogenase (PHGDH). The PHGDH product, 3-phosphohydroxypyruvate (3-PHP), is transaminated by phosphoserine aminotransferase (PSAT) to 3-phosphoserine (3-PS). 3-PS is converted to serine, catalyzed by phosphoserine phosphatase (PSPH). Serine is accumulated by mitochondria via transport by SFXN1/3. Folates are transported into mitochondria by SLC25A32. Mitochondrial THF serves as a cofactor for SHMT2 to generate glycine and 5,10-me-THF; 5,10-me-THF is subsequently converted to 10-formyl-THF (10-CHO-THF) by MTHFD2/MTHFD2L generating NAD(P)H. 10-CHO-THF is metabolized to formate and THF by MTHFD1L, generating ATP. 10-CHO-THF is also metabolized by ALDH1L2 to THF, CO_2_ and NADPH. Mitochondrial formate is transported into the cytosol where it is condensed with THF to form 10-CHO-THF catalyzed by MTHFD1. C1 units from 10-CHO-THF are incorporated into purine nucleotides in reactions catalyzed by GARFTase and AICARFTase (Fig. 2). 10-CHO-THF in the cytosol is converted to 5,10-me-THF by MTHFD1. 5,10-me-THF is reduced by 5,10-me-THF reductase to 5-methyl-THF for the synthesis of methionine and S-adenosyl methionine (SAM) by methionine synthase (MS) and methionine adenosyltransferase (MAT) required for DNA and protein methylation reactions. In the cytosol, ATP is released by ABC transporters, pannexin1 or connexins (A.P.C.), then degraded to AMP and adenosine by the cell-surface ectonucleotidases CD39 and CD73, respectively. The adenosine receptor (A2AR) binds extracellular adenosine to initiate adenosinergic signaling, which suppresses the production of interferon gamma (INFγ) in CD8^+^ T and natural killer cells, and interleukin 2 (IL-2) in CD8^+^ T cells. This results in elevation of programmed cell death protein 1 expression on CD8^+^ T cells. Extracellular adenosine also polarizes myeloid cells toward the protumor (M2) macrophages and promotes the proliferation of MDSCs. (Created in BioRender).

**Fig. 2.**
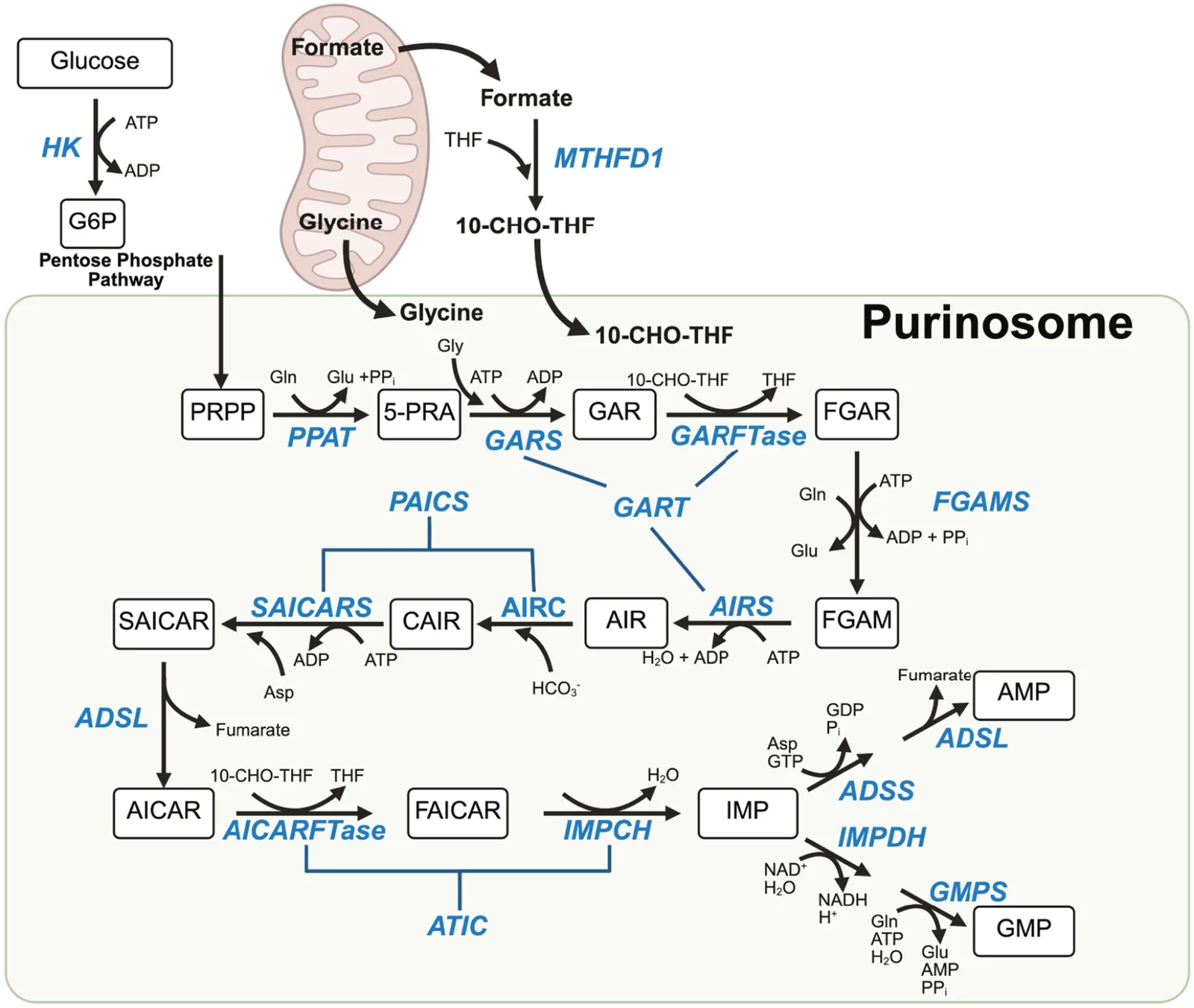
De novo purine biosynthesis. Purines are synthesized de novo in a 10-step process in the purinosome complex whereby the enzymes are located in close proximity to one another and to the mitochondria for efficient shuttling of critical metabolites.^55^ Phosphoribosyl pyrophosphate (PRPP) derived from the pentose phosphate pathway is converted to 5-phosphoribosylamine (5-PRA) by phosphoribosyl aminotransferase (PPAT). The second, third and fifth steps are catalyzed by the trifunctional glycinamide ribonucleotide transferase (GAT) enzyme. Glycine from the mitochondria is incorporated into glycinamide ribonucleotide (GAR) catalyzed by glycinamide ribonucleotide synthase (GARS). C1 units carried by 10-formyl THF (10-CHO-THF) are incorporated by GARFTase generating formyl GAR (fGAR). Formylglycinamide synthase (FGAMS) catalyzes conversion of fGAR to formylglycinamidine ribonucleotide (FGAM). The 5-aminoimidazole ribonucleotide synthase (AIRS) activity of GART converts FGAM to 5-aminoimidazole ribonucleotide (AIR) in an ATP-dependent reaction. The bifunctional phosphoribosylaminoimidazole carboxylase/succinocarboxamide synthase (PAICS) includes the aminoimidazole ribonucleotide carboxylase (AIRC) and the 4-N-succinylcarboxamide AICAR synthase (SAICARS) domains and catalyzes the sixth and seventh steps. Adenylosuccinate lyase (ADSL) generates AICAR; AICAR formyltransferase/IMP cyclohydrolase (ATIC) incorporates a C1 unit from 10-CHO-THF by the AICARFTase domain. The IMP cyclohydrolase (IMPCH) domain of ATIC catalyzes the ninth step, generating IMP, which serves as the precursor to AMP by adenylosuccinate synthase (ADSS) and ADSL or to GMP by IMP dehydrogenase (IMPDH) and GMP synthase (GMPS). (Created in BioRender).

**Table 1:** Inhibitors of C1 metabolism in cancer

Compound	Structure	Target(s)	Reference
Pemetrexed	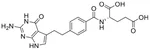	TS, DHFR, GARFTase, AICARFTase	66
AGF17, n = 1, Ar = 1′4′-phenyl AGF23, n = 2, Ar = 1′4′-phenyl AGF94, n = 1, Ar = 2′5′-thienyl AGF150, n = 1, Ar = 3′5′-thienyl AGF154, n = 1, Ar = 2′4′-thienyl AGF278, n = 2, Ar = 3′F-2′,4′-thienyl AGF291, n = 1, Ar = 1′4′-phenyl AGF320, n = 3, Ar = 2′,5′-thienyl AGF359, n = 1, Ar = 2′F-1′4′-phenyl AGF347, n =2, Ar = 2′F-1′4′-phenyl	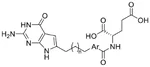	GARFTase	64,65,67,68
	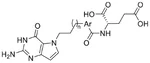	GARFTase, AICARFTase, SHMT1, SHMT2	45,69,70
SHIN1	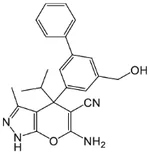	SHMT1, SHMT2	46
SHIN2	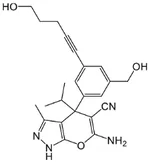	SHMT1, SHMT2	71
TH7299	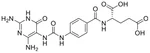	MTHFD2, MTHFD2L, MTHFD1	72
TH9028	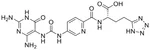	MTHFD2, MTHFD2L, MTHFD1	72
TH9619	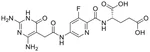	MTHFD2, MTHFD2L, MTHFD1	72,73
DS18561882	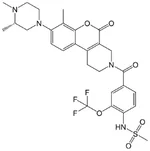	MTHFD2, MTHFD1	74
Compound 41 (chalcone derived compound)	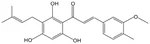	MTHFD2, MTHFD1	75

Listed are promising C1 inhibitors including structures, established intracellular targets and relevant literatures. For the AGF series of pyrrolopyrimidine antifolates (AGF17, AGF291, etc) the length of the linker (*n*) and the nature of the aromatic side-chain are summarized.

## Data Availability

This review article contains no datasets generated or analyzed during the current study.

## Abbreviations

AICAR: 5-aminoimidazole-4-carboxamide ribonucleotide
AICARFTase: 5-aminoimidazole-4-carboximide ribonucleotide formyltransferase
ALDH1L1: aldehyde dehydrogenase 1 family member L1
ALDH1L2: aldehyde dehydrogenase 1 family member L2
C1: one-carbon
DHF: dihydrofolate
DHFR: dihydrofolate reductase
FPGS: folylpolyglutamate synthetase
FR: folate receptor
GARFTase: glycinamide ribonucleotide formyltransferase
MDSC: myeloid-derived suppressor cell
me: methylene
MTHFD2L: 5,10-methylene tetrahydrofolate dehydrogenase 2-like
PCFT: proton-coupled folate transporter
PD-L1: programmed death-ligand 1
PHGDH: phosphoglycerate dehydrogenase
RFC: reduced folate carrier
ROS: reactive oxygen species
SFXN1: sideroflexin 1
SHMT2: serine hydroxymethyltransferase 2
TAM: tumor-associated macrophage
THF: tetrahydrofolate
TME: tumor microenvironment
TS: thymidylate synthase
